#  LncRNA MRAK048635_P1 is critical for vascular smooth muscle cell function and phenotypic switching in essential hypertension

**DOI:** 10.1042/BSR20182229

**Published:** 2019-03-19

**Authors:** Genqiang Fang, Jia Qi, Liya Huang, Xianxian Zhao

**Affiliations:** 1 Department of Cardiovasology, Changhai Hospital Affiliated to Second Military Medical University, Shanghai 200433, China; 2 Department of Geriatric Medicine, Xinhua Hospital, School of Medicine, Shanghai Jiao Tong University, Shanghai 200092, China; 3 Department of Pharmacy, Xinhua Hospital, School of Medicine, Shanghai Jiao Tong University, Shanghai 200092, China

**Keywords:** essential hypertension, long noncoding RNA (lncRNA), phenotypic switching, vascular smooth muscle cells, vascular remodeling

## Abstract

Vascular remodeling caused by essential hypertension is a leading cause of death in patients, and vascular smooth muscle cell (VSMC) dysfunction and phenotypic switching result in vascular remodeling. Therefore, inhibiting cell dysfunction and phenotypic switching in VSMCs may be a new treatment strategy for essential hypertension. The aim of the current study is to explore the roles of long non-coding RNA (lncRNA) MRAK048635_P1 in VSMC function and phenotypic switching. The MRAK048635_P1 level was determined in spontaneously hypertensive rats (SHRs) and VSMCs isolated from SHRs. MRAK048635_P1 was knocked down using a specific siRNA in VSMCs isolated from the thoracic aorta of SHRs and Wistar–Kyoto rats. Then, the proliferation and migration of VSMCs were determined using a cell counting kit-8 (CCK-8), a ^3^H labeling method, a transwell assay, and a wound healing assay. Flow cytometry was used to test the effect of MRAK048635_P1 on VSMC apoptosis. The protein and mRNA levels of associated genes were measured through Western blotting, immunofluorescence, and Quantitative Reverse Transcription-Polymerase Chain Reaction (qRT-PCR). MRAK048635_P1 showed low expression during hypertension *in vivo* and *in vitro*. Down-regulation of lncRNA MRAK048635_P1 promoted proliferation and migration and inhibited apoptosis in VSMCs isolated from healthy rat vascular tissue and SHR-derived VSMCs. Importantly, we also found that down-regulation of MRAK048635_P1 could induce VSMC phenotypic switching from a contractile to a secretory phenotype. In conclusion, our findings reveal that decreased MRAK048635_P1 is probably an important factor for vascular remodeling by affecting VSMC cell function and phenotypic switching in essential hypertension.

## Introduction

Essential hypertension is a clinical syndrome characterized by increased systemic arterial blood pressure (systolic pressure ≥ 140 mmHg and/or diastolic pressure ≥ 90 mmHg), accompanied by functional or organic damage to the heart, brain, kidney, and other organs [1]. Hypertension is the most important risk factor for cardiovascular and cerebrovascular diseases. A continuous increase in blood pressure leads to vascular remodeling and vascular dysfunction, which is the leading cause of death in patients [2,3]. Previous studies have shown that abnormal proliferation, migration, invasion, and phenotypic switching of vascular smooth muscle (VSM) cells (VSMCs) are important causes of vascular remodeling [4,5]. Therefore, inhibiting cell dysfunction and phenotypic switching in VSMCs may represent a new treatment strategy for essential hypertension.

Long non-coding RNA (lncRNA) greater than 200 nts in length is a class of important non-coding transcripts [6]. LncRNAs can regulate the expression of target genes at the transcriptional and post-transcriptional levels, and widely participate in biological processes, such as the maintenance of nervous system function and stem cell pluripotency [7,8]. Additionally, lncRNA is also closely related to the initiation and progression of malignant tumors [9]. Recently, lncRNA has also been reported to play a crucial role in cardiovascular diseases. Kumarswamy et al. [10] found that the expression of lncRNA uc022bqs.1 was increased in patients with heart failure and was associated with a higher risk of cardiovascular death. Liu et al. [11] found that lncRNA metastasis associated lung adenocarcinoma transcript 1 (MALAT1) is closely related to diabetes-induced microvascular dysfunction. Notably, previous studies also indicated that lncRNA may play a role in regulating blood pressure. Chen et al. [12] reported that high expression of lncRNA NR_104181 and low expression of NR_027032 were possibly related to the risk of hypertension. However, studies on the expression profile of lncRNA in VSMCs in hypertension are still scarce. Additionally, data concerning the effect and mechanism of specific lncRNAs in the proliferation, apoptosis, migration, and phenotypic switching of VSMCs are also rare.

To explore the biological role of lncRNAs in VSMCs in hypertension, Yao et al. [13] screened lncRNA expression profiles in the aortas of spontaneously hypertensive rats (SHRs) and Wistar–Kyoto rats (WKYs) using a gene microarray, and the results showed that 68 lncRNAs were up-regulated and 167 lncRNAs were down-regulated in the SHR aorta. The researchers also found that lncRNA XR007793 was involved in regulating the proliferation and migration of VSMCs [13]. We also noticed significant differences in the expression level of MRAK048635_P1 in SHR compared with WKY, but the relationship between MRAK048635_P1 and VSMCs has not yet been reported [13]. Therefore, MRAK048635_P1 provided a starting point for the current research.

## Materials and methods

### Preparations for animal model and VSMCs

Male 3-month-old SHR and WKY rats weighing 280–330 g were purchased from Shanghai Experimental Animal Center, Chinese Academy of Science. VSMCs were isolated from the aortas of SHR and WKY rats, using the method described by Shi et al. [14]. All animal experiments were performed with the approval of the animal ethics committee of Xinhua Hospital, School of Medicine, Shanghai Jiao Tong University, and followed the principles of laboratory animal care.

### siRNA transfection

si-MRAK048635_P1 and scrambled siRNA plasmids were synthesized by Gene Pharma (Shanghai, China). These plasmids, at a concentration of 10 nM, were transfected into VSMCs using Lipofectamine RNAiMAX Transfection Reagent (catalog number: 13-778-075, Invitrogen) in accordance with the manufacturer’s instructions. After 24 h, the cells were seeded into 24-well plates, and allowed to grow for another 24–48 h before the next experiment. The specific siRNA primers were: siRNA 1 (sense: 5′-GCAGCCACUUACAUAGAAUTT-3′ and antisense: 5′-AUUCUAUGUAAGUGGCUGCTT-3′); siRNA 2 (sense: 5′-GGACUUACGAAAGGACAAATT-3′ and antisense: 5′-UUUGUCCUUUCGUAAGUCCTT-3′); siRNA 3 (sense: 5′-CCAGAAAUUAUCCAGUAAATT-3′ and antisense: 5′-UUUACUGGAUAAUUUCUGGTT-3′); scrambled siRNA (sense: 5′-UUCUCCGAACGUGUCACGUTT-3′ and antisense: 5′-ACGUGACACGUUCGGAGAATT-3′).

### Serum extraction

Blood samples were collected and coagulated for 1–2 h at 37°C (without anticoagulant). After storage overnight at 4°C, the serum was separated through centrifugation at 3000 rpm at 4°C for 10 min and stored at −80°C until further use.

### Fluorescence *in situ* hybridization

The subcellular localization of MRAK048635_P1 was detected using fluorescence *in situ* hybridization (FISH) in accordance with the instructions provided with a FISH kit (Ribobio Co.). In brief, VSMCs were fixed using 4% paraformaldehyde for 10 min at room temperature. Prehybridization was performed using a lncRNA FISH probe mix at 42°C for 1 h. After that, hybridization was performed by adding a MRAK048635_P1 FISH probe mix and incubating the samples overnight at 42°C. After washing using 2× saline sodium citrate (SSC), the cell nuclei were stained using DAPI. Finally, the samples were observed using a fluorescence microscope (Olympus, Japan).

### Measurement of cell proliferation and cell migration

Cell proliferation was measured using a cell counting kit-8 (CCK-8) (Dojindo, ck-04) and an ^3^H labeling method (GENMED, GMS11022.2) following the manufacturer’s instructions. Cell migration was measured through a wound healing assay and transwell assay.

### Flow cytometry

VSMC apoptosis was measured using an FITC apoptosis detection kit (BioVision, U.S.A.). VSMCs were transfected and 48 h later collected in 5 ml tubes. After washing in ice-cold PBS, cells in 10 mM HEPES/NaOH were resuspended in Annexin V binding buffer. Then 5 ml of FITC-Annexin V was added into the cell suspension. After propidium iodide staining, the cells were analyzed through flow cytometry (CytoFLEX LX, Beckman).

### Quantitative Reverse Transcription-Polymerase Chain Reaction (qRT-PCR)

Total RNA was extracted using TRIzol reagent. cDNA was synthesized using All-in-One cDNA Synthesis SuperMix (Biotool, B24403), and qPCR was performed using 2× SYBR Green qPCR Master Mix (Biotool, B21202). The specific primers were: MRAK048635_P1 (forward: 5′-CACTCTTGTCTGGGGATGGTG-3′, reverse: 5′-GCAGTCACTTGAGAAATGCCC-3′); alpha smooth muscle actin (α-SMA) (forward: 5′-ACCATCGGGAATGAACGCTT-3′ and reverse: 5′-CTGTCAGCAATGCCTGGGTA-3′); smooth muscle 22 alpha (SM22α) (forward: 5′-ATCCTATGGCATGAGCCGTG-3′ and reverse: 5′-CAGGCTGTTCACCAACTTGC-3′); calponin (forward: 5′-CTGCCTGACCCCGGAATATC-3′ and reverse: 5′-GGCCTGATCTCCCCAAACTG-3′); osteopontin (forward: 5′-CCAGCCAAGGACCAACTACA-3′ and reverse: 5′-CCAAGTGGCTACAGCATCTGA-3′); β-actin (forward: 5′-GCGCAAGTACTCTGTGTGGA-3′ and reverse: 5′-AGGGTGTAAAACGCAGCTCAG-3′).

### Western blotting and immunofluorescence

For Western blotting, protein concentrations in VSMC lysates were quantitated using a BCA Protein Assay Kit (Thermo Fisher Scientific, No. 23225). Proteins (30 μg) were separated through 15% SDS/PAGE and then transferred on to PVDF membranes (Millipore). After 1 h incubation in 5% non-fat milk at room temperature, the blots were further incubated with primary antibodies overnight at 4°C. The primary antibodies used were: anti-Cyclin D1 (Abcam, ab134175), anti-Cyclin E (Abcam, ab33911), anti-cyclin-dependent kinase (CDK) 2 (CDK2) (Abcam, ab32147), anti-CDK4 (Abcam, ab108357), anti-Rb (phospho S807) (Abcam, ab184796), anti-β actin (HC-101-02), anti-active caspase3 (Abcam, ab2302), anti-cleaved poly ADP-ribose polymerase (PARP) (Abcam, ab4830), anti-α-SMA (Abcam, ab32575), anti-SM22α (Abcam, ab14106), anti-Calponin (Abcam, ab46794), anti-Osteopontin (Abcam, ab8448), anti-smoothelin (Abcam, ab21108), and anti-tropomyosin (Abcam, ab181085).

For immunofluorescence, VSMCs were incubated in primary antibodies against α-SMA (Abcam, ab32575) or calponin (Abcam, ab46794).

### Statistical analysis

All values are expressed as mean ± S.D. The differences between two groups were analyzed through a one-way ANOVA using GraphPad Prism 5.0. *P<0.05 was considered to be statistically significant.

## Results

### MRAK048635_P1 is down-regulated in SHRs and VSMCs from SHRs

**Figure 1:**
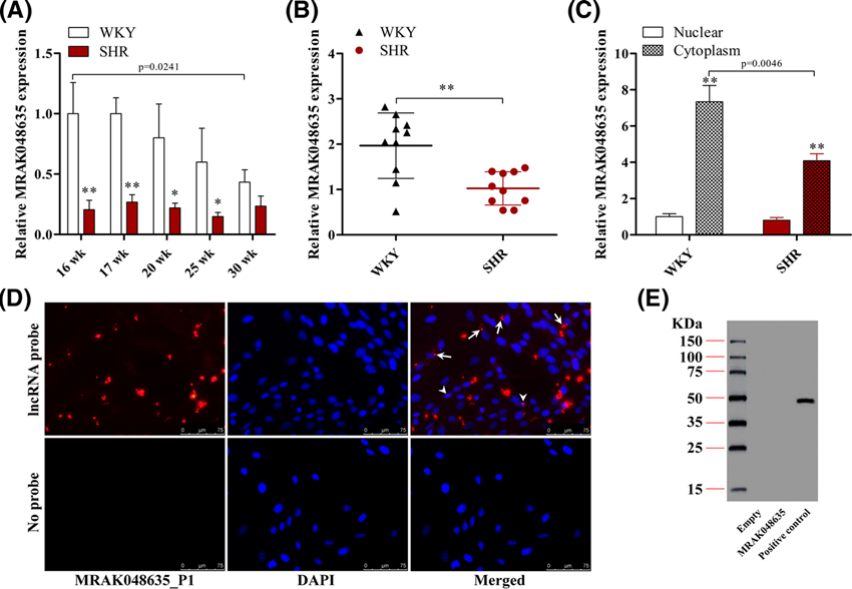
MRAK048635_P1 is down-regulated in hypertension (**A**) The expression of MRAK048635_P1 was detected using qRT-PCR in VSMCs isolated from SHR (*n*=15) and WKY (*n*=15) rats of various ages. *P<0.05, **P<0.01, compared with WKY rats. (**B**) The expression of MRAK048635_P1 was detected using qRT-PCR in serum isolated from SHR (*n*=10) and WKY (*n*=10) rats. **P<0.01, compared with WKY rats. (**C**) The expression of MRAK048635_P1 was detected using qRT-PCR in VSMCs isolated from SHR and WKY rats. **P<0.01, compared with nuclear. The data were representative of three independent experiments with ten rats per group. (**D**) The subcellular location of MRAK048635_P1 was detected using FISH; arrows indicate cytoplasm, and arrow heads indicate nuclear. (**E**) Western blotting was used to detect the protein coding function of MRAK048635_P1. LncRNA was cloned into an adenoviral vector and expressed using the TnT Quick Coupled Transcription/Translation System (Promega). Luciferase *in vitro* translation served as positive control. Empty stands for empty vector. Abbreviation: qRT-PCR, Quantitative Reverse Transcription-Polymerase Chain Reaction.

VSM isolated from SHRs was used to determine the expression level of MRAK048635_P1 in hypertension suppression. As shown in Figure 1A, MRAK048635_P1 showed low expression in SHR tissue compared with WKY tissue. We also found that there is a low level of MRAK048635_P1 in SHR serum (Figure 1B). At the cellular level, low nuclear expression of MRAK048635_P1 was observed in both SHR and WKY samples, but the expression of MRAK048635_P1 was lower in cytoplasm in SHR samples compared with WKY samples (Figure 1C). Next, FISH was performed to measure the subcellular location of MRAK048635_P1, and this revealed that MRAK048635_P1 was mainly expressed in the cytoplasm (Figure 1D). Moreover, Western blot analysis revealed that MRAK048635_P1 did encode a polypeptide chain (Figure 1E). Taken together, these results indicated that MRAK048635_P1 is down-regulated in SHR thoracic aorta tissues and the corresponding VSMCs.

### MRAK048635_P1 knockdown promotes the proliferation and migration of VSMCs from WKY rats

**Figure 2:**
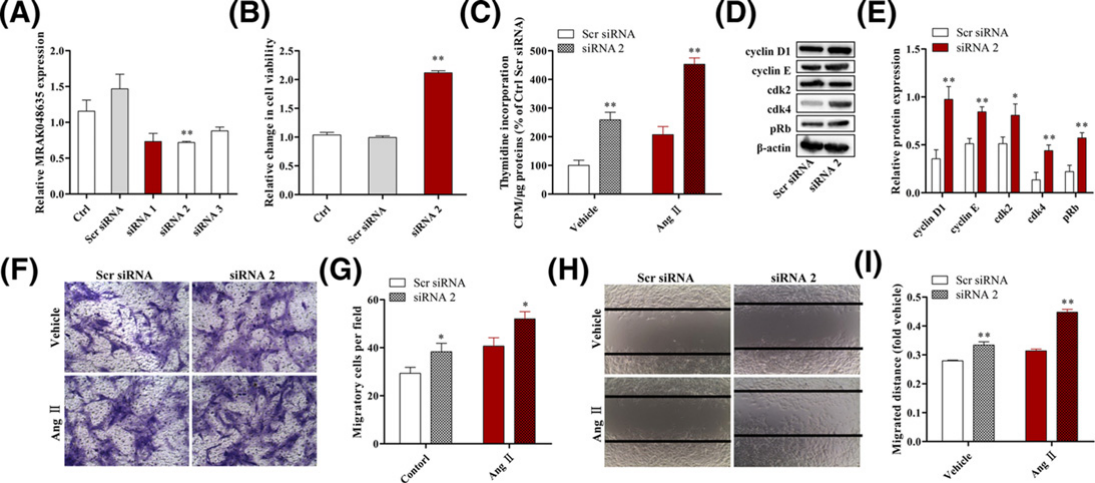
Down-regulation of MRAK048635_P1 promotes the proliferation and migration of VSMCs (**A**) The silencing efficacy of MRAK048635_P1 was measured using qRT-PCR in VSMCs. The data were representative of three independent experiments with five rats per group. (**B**) CCK-8 was used to detect the viability of VSMCs transfected with siR-lncRNA MRAK048635_P1. The data were representative of three independent experiments with five rats per group. (**C**) The ^3^H labeling method was used to detect the viability of VSMCs transfected with siR-MRAK048635_P1. The data were representative of three independent experiments with five rats per group. (**D,E**) The protein expression of Cyclin D1, Cyclin E, CDK2, CDK4, and p-Rb in VSMCs transfected with siR-MRAK048635_P1, and their semi-quantitative results in the histogram. (**F**,**G**) A transwell assay was performed to analyze the migration of VSMCs transfected using siR-MRAK048635_P1 or Scr siRNA. (**H**,**I**) A wound healing assay was performed to analyze the migration of VSMCs transfected using siR-MRAK048635_P1 or Scr siRNA. *P<0.05, **P<0.01, compared with Scr siRNA. Abbreviation: qRT-PCR, Quantitative Reverse Transcription-Polymerase Chain Reaction.

To examine the effect of MRAK048635_P1 on the proliferative and migratory abilities of VSMCs, siRNA was transfected into VSMCs isolated from healthy rat arterial tissue to specifically down-regulate the expression of MRAK048635_P1 (Figure 2A). A CCK-8 assay showed that cell viability was significantly enhanced after knocking down MRAK048635_P1 (Figure 2B). In addition, data from ^3^H labeling study indicated that the down-regulation of MRAK048635_P1 enhanced the proliferation of VSMCs in SHRs (Figure 2C). Angiotensin II (Ang II) is a potent endogenously occurring vasoconstrictor. VSMCs treated using Ang II can closely approximate the hypertensive state *in vitro*. As depicted in Figure 2C, the proliferative viability of VSMCs was also promoted by down-regulation of MRAK048635_P1 during simulated hypertension status *in vitro*.

The mechanism underlying MRAK048635_P1-mediated regulation of VSMC proliferation was still unclear. To address this, we tested some well-known markers of cell-cycle activities: cyclinD1/E, CDK2/4, and phosphorylated retinoblastoma gene (p-*Rb*). As shown in Figure 2D,E, knocking down MRAK048635_P1 promoted the expression of these cycle-related markers, suggesting that low level of MRAK048635_P1 might enhance the proliferation of VSMCs via regulating cyclinD1/E, CDK2/4, and p-*Rb* in hypertension (Figure 2D,E).

Having confirmed the effect of MRAK048635_P1 siRNA on VSMC proliferation, we next explored its function in VSMC migration. As shown in Figure 2F,G, the number of VSMCs that passed through a transwell membrane was significantly increased after knocking down MRAK048635_P1 under physiological and pathological conditions (with or without Ang II). Additionally, the results from a cell wound healing assay showed that down-regulation of MRAK048635_P1 could increase the migratory distance of VSMCs compared with the control group (Figure 2H,I).

### MRAK048635_P1 knockdown inhibits the apoptosis of VSMCs from WKY rats

**Figure 3:**
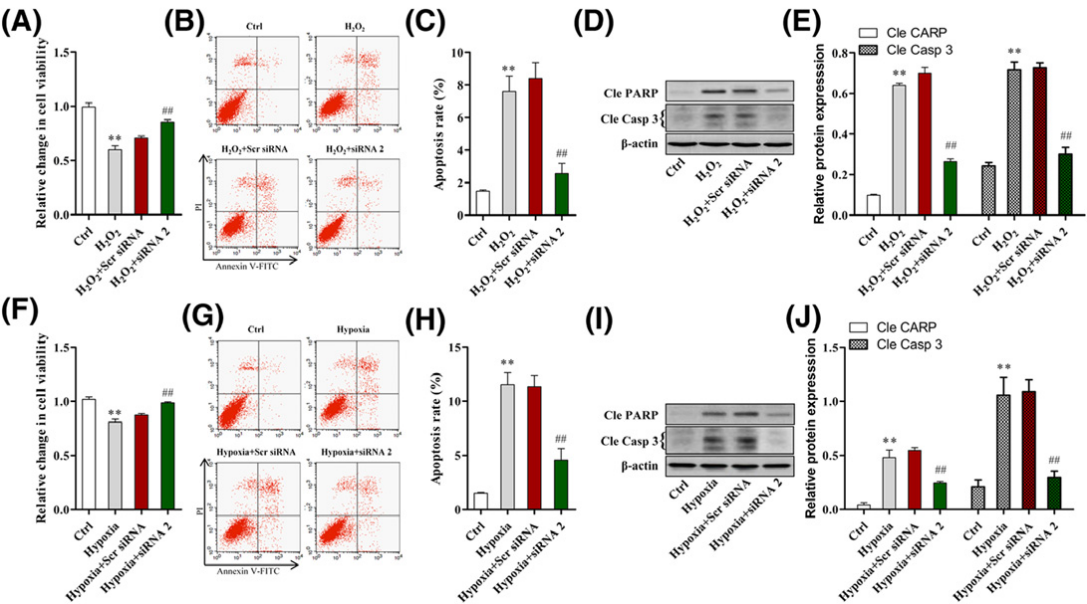
Down-regulation of MRAK048635_P1 inhibits the apoptosis of VSMCs (**A**) CCK-8 was performed to analyze H_2_O_2_-mediated apoptosis in VSMCs transfected using siR-MRAK048635_P1. The data were representative of three independent experiments with five rats per group. (**B**,**C**) Flow cytometry was performed to analyze the apoptosis of VSMCs transfected using siR-MRAK048635_P1 or Scr siRNA in the presence or absence of H_2_O_2_. (**D**,**E**) Western blot analysis of cleaved PARP and cleaved caspase 3 after transfection with siR-MRAK048635_P1 or Scr siRNA with or without H_2_O_2_. (**F**) CCK-8 was performed to analyze hypoxia-mediated apoptosis in VSMCs transfected using siR-MRAK048635_P1. The data were representative of three independent experiments with five rats per group. (**G**,**H**) Flow cytometry was performed to analyze the apoptosis of VSMCs transfected using siR-MRAK048635_P1 or Scr siRNAs. (**I**,**J**) Western blot analysis of cleaved PARP and cleaved caspase 3 after transfection with siR-MRAK048635_P1 or Scr siRNA with or without H_2_O_2_. **P<0.01, compared with control; ^##^*P*<0.01, compared with Scramble siRNA treatment.

As shown in Figure 3A, VSMCs were treated using H_2_O_2_ to induce significantly suppressed viability. However, we found that cell viability could be rescued by down-regulation of MRAK048635_P1 (Figure 3A). Next, we assessed VSMC apoptosis using flow cytometry. The results showed that the apoptosis rate of VSMCs treated using H_2_O_2_ increased significantly; however, this effect could be rescued by knocking down MRAK048635_P1 (Figure 3B,C). To further confirm the apoptosis-induced effects of MRAK048635_P1 on VSMCs, the protein expression of cleaved caspase3 and cleaved PARP were detected using Western blotting. The results showed that the protein levels of cleaved caspase3 and cleaved PARP after treating with H_2_O_2_ increased significantly; similarly, this effect also could be rescued by knocking down MRAK048635_P1 (Figure 3D,E). Moreover, we also found that inhibition of MRAK048635_P1 restored hypoxia-mediated apoptosis and the increase in cleaved caspase3 and cleaved PARP induced by hypoxia in VSMCs (Figure 3F–J).

### MRAK048635_P1 knockdown promotes phenotypic switching of VSMCs from WKY rats

**Figure 4:**
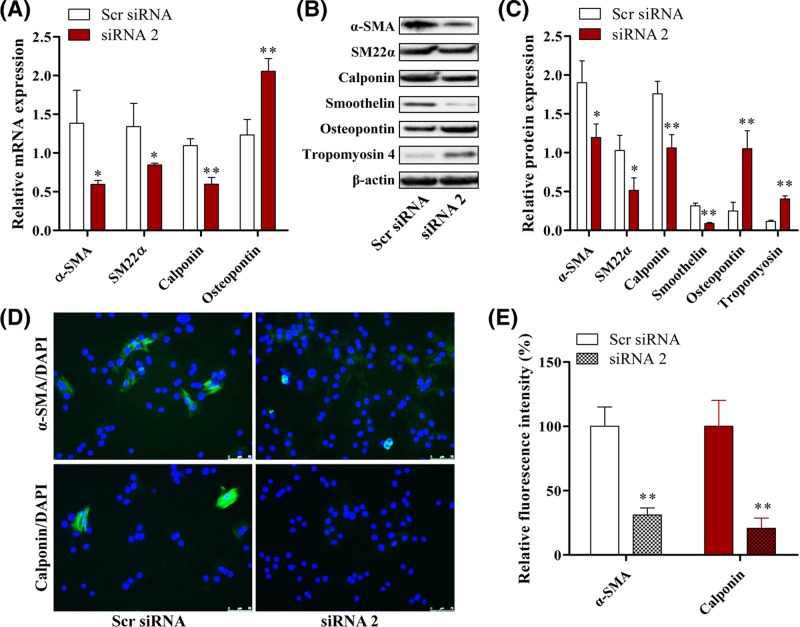
Down-regulation of MRAK048635_P1 promotes phenotypic switching of VSMCs (**A**) The mRNA expression of α-SMA, SM22α, calponin and osteopontin in VSMCs transfected with siR-MRAK048635_P1 compared with Scr siRNA. The data were representative of three independent experiments with five rats per group. (**B**,**C**) The protein expression of α-SMA, SM22α, calponin, and osteopontin in VSMCs transfected with siR-MRAK048635_P1 compared with Scr siRNA. (**D**,**E**) Immunofluorescence showed the expression of α-SMA and calponin in VSMCs transfected with siR-MRAK048635_P1, and quantitated using ImagePro Plus software. *P<0.05, **P<0.01, compared with Scr siRNA.

VSMC phenotypic switching from a contractile to a secretory phenotype is required for vascular remodeling. To further explore the mechanism through which MRAK048635_P1 regulates VSMC function, we tested six phenotypic markers (four contractile phenotype markers: α-SMA, SM22α, calponin, and smoothelin; two secretory phenotype markers: osteopontin and tropomyosin 4) after knocking down MRAK048635_P1 in VSMCs. As shown in Figure 4A, the mRNA levels of α-SMA, SM22α, and calponin were significantly reduced whereas osteopontin mRNA levels were enhanced by down-regulation of MRAK048635_P1, compared with the control group. Western blot analysis also showed that the protein expression of α-SMA, SM22α, calponin, and smoothelin was inhibited, while the protein expression of osteopontin and tropomyosin 4 was enhanced after knocking down MRAK048635_P1, suggesting that down-regulation of MRAK048635_P1 could induce phenotypic switching of VSMCs from a contractile to a secretory phenotype (Figure 4B,C).

VSMC contractile phenotypic markers (α-SMA and calponin) were further assessed via immunofluorescence after knocking down MRAK048635_P1. Compared with the control group, the expression of α-SMA and calponin was significantly inhibited in the MRAK048635_P1 knockdown group (Figure 4D,E).

### MRAK048635_P1 knockdown promotes the proliferation, migration, and phenotypic switching of SHR-derived VSMCs, and inhibits their apoptosis

**Figure 5:**
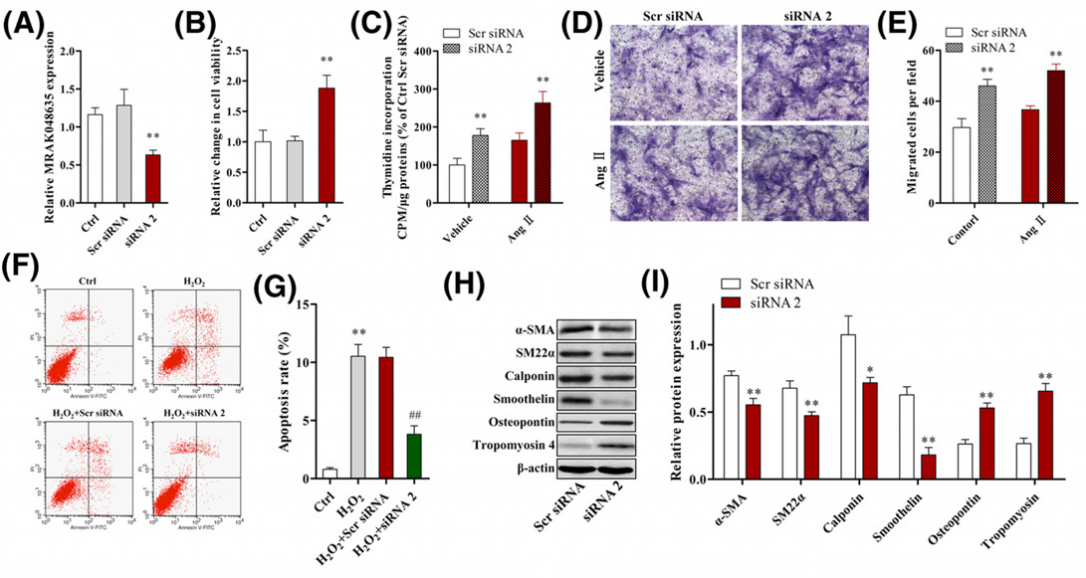
MRAK048635_P1 knockdown promotes the proliferation, migration, and phenotypic modulation of SHR-derived VSMCs, and inhibits their apoptosis (**A**) The silencing efficacy of MRAK048635_P1 was measured through qRT-PCR in SHR-derived VSMCs. **P<0.01, compared with Scr siRNA. The data were representative of three independent experiments with five rats per group. (**B**) CCK-8 assay was used to detect the viability of SHR-derived VSMCs transfected with siRMRAK048635_P1. **P<0.01, compared with Scr siRNA. The data were representative of three independent experiments with five rats per group. (**C**) The ^3^H labeling method was used to detect the viability of SHR-derived VSMCs transfected with siR-MRAK048635_P1. **P<0.01, compared with Scr siRNA. The data were representative of three independent experiments with five rats per group. (**D**,**E**) A transwell assay was performed to analyze the migration of SHR-derived VSMCs transfected using siR-MRAK048635_P1 or Scr siRNA. *P<0.05, compared with Scr siRNA. (**F**,**G**) Flow cytometry was performed to analyze apoptosis in SHR-derived VSMCs transfected using siR-MRAK048635_P1 or Scr siRNA. **P<0.01, compared with control group; ^##^*P*<0.01, compared with H_2_O_2_+Scr siRNA. (**H**,**I**) The protein expression of α-SMA, SM22α, calponin, and osteopontin in SHR-derived VSMCs transfected with siR-MRAK048635_P1 and the semi-quantitative results. *P<0.05, **P<0.01, compared with Scr siRNA. Abbreviation: qRT-PCR, Quantitative Reverse Transcription-Polymerase Chain Reaction.

siRNA was transfected into SHR-derived VSMCs to specifically down-regulate the expression of MRAK048635_P1 (Figure 5A). As shown in Figure 5B,C, CCK-8 and ^3^H labeling tests indicated that cell proliferation was significantly enhanced after knocking down MRAK048635_P1 in SHR-derived VSMCs. Moreover, the proliferative potential of SHR-derived VSMCs treated using Ang II was also promoted by down-regulation of MRAK048635_P1 during simulated hypertension status *in vitro* (Figure 5C). The results from a transwell assay showed that down-regulation of MRAK048635_P1 could increase the number of SHR-derived VSMCs that passed through a transwell membrane under physiological and pathological conditions (with or without Ang II) (Figure 5D,E). Moreover, we also found that inhibition of MRAK048635_P1 rescued H_2_O_2_-mediated apoptosis in SHR-derived VSMCs (Figure 5F,G).

We then tested some phenotypic markers after knocking down MRAK048635_P1 in SHR-derived VSMCs. Western blot analysis indicated that the protein expression of contractile phenotype markers (α-SMA, SM22α, smoothelin, and calponin) decreased, while the protein levels of osteopontin and tropomyosin4 were increased after knocking down MRAK048635_P1, suggesting that down-regulation of MRAK048635_P1 could also induce phenotypic switching of SHR-derived VSMCs from a contractile to a secretory phenotype (Figure 5H,I).

## Discussion

Hypertension is the most common risk factor for cardiovascular disease and death [1]. In recent years, the treatment for patients with hypertension has progressed because of the application of new drugs [15,16]. Nevertheless, there are still two major problems in the prevention and treatment of hypertension. One is the primary and secondary prevention of hypertension. Currently, health assessments cannot accurately predict the ‘tendency’ of hypertension, and the detection rate of hypertension is not high [17]. Second, essential hypertension is caused by a combination of genetic and environmental factors, but the exact molecular mechanisms underlying its initiation and development are still not very clear, which is also the fundamental reason why essential hypertension cannot be cured at present [18].

Vascular remodeling is a characteristic pathological feature of hypertension [19]. The main mechanism of vascular remodeling is the activation of silent VSMCs in a physiological state, which leads to abnormal VSMC proliferation and the formation of neointima [20]. Therefore, exploring the pathophysiological mechanism underlying VSMC proliferation is the key to resolving the problem of vascular remodeling. However, the specific mechanism through which VSMCs in a silent state are activated into a proliferative state remains unclear in essential hypertension.

LncRNA includes small nucleolar RNA, enhancer RNA, and intergenic transcripts. Numerous studies have reported that lncRNAs are involved in a variety of biological processes and disease progression, such as cell proliferation, apoptosis and differentiation, genomic imprinting, tumor progression, neurodegeneration, and metabolic diseases [21,22]. Recent studies have shown that lncRNAs can sense changes in blood vessel transmural pressure, and therefore play an important role in the regulation of VSMC function [23]. Therefore, studying the function and regulatory mechanisms of lncRNAs is beneficial for the treatment of essential hypertension.

In the present study, we found that MRAK048635_P1 is down-regulated in SHRs and VSMCs isolated from SHRs, indicating the potential roles of MRAK048635_P1 in essential hypertension (Figure 1). Additionally, we also noticed that the level of MRAK048635_P1 in WKY rats aged over 30 weeks was down-regulated. Previous studies have reported that WKY rats show higher blood pressure with ageing, and decreasing MRAK048635_P1 levels might account for, or at least be associated with, this phenomenon [24–26]. Vascular remodeling is a characteristic pathological feature of hypertension, and the proliferation and subendothelial migration of VSMCs are central components of vascular remodeling. The present study confirmed that MRAK048635_P1 siRNA could enhance the proliferative and migratory ability of VSMCs (Figure 2). CDKs are Ser/Thr kinases, and can bind to cyclin D1/E to form a heterodimer and further activate the cell cycle from G_1_ phase to S phase [27]. It has been shown that lncRNA can promote the progression of several malignancies by regulating CDK2/4 [28,29]. Similarly, the current findings revealed that knocking down MRAK048635_P1 in VSMCs resulted in the overexpression of cyclin D1, cyclin E, CDK2, and CDK4, indicating that MRAK048635_P1 knockdown might promote VSMC proliferation by targetting these cycle-related factors (Figure 2B). Additionally, in the G_1_ phase, the Rb protein is phosphorylated by Cyclin D/CDK4 or Cyclin E/CDK2, and then the transcription factor E2F is activated to promote the transcription of proliferation-related genes [30]. Our results also showed that the level of p-*Rb* was increased after down-regulation of MRAK048635_P1 (Figure 2B).

Having demonstrated that MRAK048635_P1 interference significantly promoted VSMC proliferation and migration, we then explored the role of MRAK048635_P1 siRNA in VSMC apoptosis. As shown in Figure 3, the results revealed that MRAK048635_P1 siRNA could rescue WKY-derived VSMCs from apoptosis. In the VSMCs from SHRs, interfered MRAK048635_P1 can facilitate proliferation and migration under the same conditions [31,32]. In the present study, we also confirmed that knockdown of MRAK048635_P1 in SHR-derived VSMCs can inhibit apoptosis. These findings suggested that MRAK048635_P1 is critical for VSMCs, and the suppressed MRAK048635_P1 in essential hypertension may be a potential therapeutic target (Figure 5).

The initiation of vascular remodeling is mainly dependent upon VSMC phenotypic switching from a contractile to a secretory phenotype. Yan et al. [33] reported that the phenotypic transformation and cell proliferation of VSMCs can induce extracellular matrix (ECM) deposition in the vascular wall during intimal hyperplasia. Frismantiene et al. [34] found that in response to vascular injury, VSMCs can switch from a contractile phenotype to a secretory phenotype characterized by increased proliferation, migration, and ECM deposition. We have confirmed that MRAK048635_P1 is critical for cell function in VSMCs. The role of MRAK048635_P1 in VSMC phenotypic modulation was also determined. α-SMA, SM22α, calponin, and smoothelin, highly expressed in contractile cells and lowly expressed in secretory cells, are early specific markers of VSMC differentiation [35]. Yu et al. [36] reported that VSMCs with a contractile phenotype do not have the ability to proliferate and migrate. Osteopontin is a marker of the secretory phenotype. It is absent from healthy arteries, but demonstrates increased expression in atherosclerosis [37]. As expected, we found that the mRNA and protein levels of α-SMA, SM22α, calponin, and smoothelin were reduced whereas the expression of osteopontin was increased after knocking down MRAK048635_P1 in VSMCs isolated from healthy rat vascular tissue and SHR-derived VSMCs. In addition, tropomyosin 4 is also a marker of the secretory phenotype. Our data also showed that the expression of tropomyosin 4 was increased after knocking down MRAK048635_P1 in SHR-derived VSMCs. Taken together, these results indicated that down-regulation of MRAK048635_P1 can induce VSMC phenotypic switching from a contractile to a secretory phenotype. Furthermore, it should be noted that in order to further explore the potential mechanistic functioning of MRAK048635_P1, we attempted to predict miRNAs that might interact with MRAK048635_P1, but did not obtain relevant results. Therefore, in our future research, we plan to use high-throughput sequencing to predict miRNAs interacting with MRAK048635_P1 and their target genes.

In conclusion, our findings reveal that decreased level of lncRNA MRAK048635_P1 may be an important factor for vascular remodeling in hypertension, because it is probably capable of regulating VSMC function and phenotype. The findings in the present study suggest that decreased MRAK048635_P1 is a potential therapeutic target for essential hypertension.

## Abbreviations

Ang II: angiotensin II
CCK-8: cell counting kit-8
CDK: cyclin-dependent kinase
ECM: extracellular matrix
FISH: fluorescence *in situ* hybridization
lncRNA: long non-coding RNA
PARP: poly ADP-ribose polymerase
qRT-PCR: Quantitative Reverse Transcription-Polymerase Chain Reaction
SHR: spontaneously hypertensive rat
SM22α: smooth muscle 22 alpha
SSC: saline sodium citrate
VSM: vascular smooth muscle
VSMC: VSM cell
MALAT1: metastasis associated lung adenocarcinoma transcript 1
α-SMA: alpha smooth muscle actin
